# Advice to future family physicians: findings from qualitative interviews with family medicine residents and early-career family physicians

**DOI:** 10.1186/s12909-024-05882-5

**Published:** 2024-08-20

**Authors:** Sean Wang, Richard Buote, Lauren R. Moritz, M. Ruth Lavergne, Emily Gard Marshall

**Affiliations:** Primary Care Research Unit, Dalhousie Family Medicine, Dalhousie University, 1465 Brenton Street Suite 402, Halifax, NS B3J 3T4 Canada

**Keywords:** Graduate medical education, Health workforce, Family medicine, Primary health care, Advice

## Abstract

**Background:**

Canadians continue to report challenges accessing primary care. Practice choices made by primary care providers shape services available to Canadians. Although there is literature observing family medicine practice trends, there is less clarity on the reasoning underlying primary care providers’ practice intentions. Advice offered by residents and early-career family physicians may reveal challenges they have experienced, how they have adapted to them, and strategies for new residents. In this paper, we examine advice family medicine residents and early-career family physicians would give to new family medicine residents.

**Methods:**

Sixty early-career family physicians and thirty residents were interviewed as part of a mixed-methods study of practice patterns of family medicine providers in Canada. During qualitative interviews, participants were asked, *“what advice would you give [a new family medicine resident] about planning their career as a family physician?”* We inductively analyzed responses to this question.

**Results:**

Advice consisted of understanding the current climate of family medicine (need for specialization, business management burden, physician burnout) and revealed reasons behind said challenges (lack of support for comprehensive clinic care, practical limitations of different practice models, and how payment models influence work-life balance). Subtheme analyses showed early-career family physicians being more vocal on understanding practical aspects of the field including practice logistics and achieving job security.

**Conclusion:**

Most advice mirrored current changes and challenges as well as revealing strategies on how primary care providers are handling the realities of practicing family medicine. Multi-modal systemic interventions may be needed to support family physicians throughout the changing reality of family medicine and ensure family medicine is an appealing specialty.

**Supplementary Information:**

The online version contains supplementary material available at 10.1186/s12909-024-05882-5.

## Background

Primary care access is essential to ensure good population health outcomes and reduce health disparities [1]. Within the Canadian health system, primary care providers, such as family physicians, are the first point of contact, acting as “gatekeepers” to other aspects of the healthcare system, including specialist care [2–4]. However, Canadians increasingly report challenges accessing primary care, shown by a steady increase in the number of Canadians without a regular primary care provider [5, 6]. Strains on primary care appear to have worsened during the COVID-19 pandemic, with higher numbers of family physicians leaving their practice during the pandemic compared to pre-pandemic. [7] There is a need to understand what is driving challenges in primary care access and influencing the choices made by primary care providers.

Over the past several decades, there have been substantial changes in practice patterns among family physicians. Compared to 1997/1998, today’s practicing family physicians have fewer contacts with patients, and fewer family physicians provide comprehensive primary care [8–10]. A variety of factors (e.g., health policy, patient population, practice setting, advice received as a trainee) [11, 12] may influence these practice patterns. While we have examined current practice trends in family medicine, there is less clarity on how and why primary care providers are adapting to the practice environment in different ways. The experiences of family medicine residents and early-career family physicians and the advice they would offer to people entering residency programs may provide insight into factors shaping practice intentions and choices and, by extension, access to primary care [13].

Advice given by experienced family physicians to trainees at pivotal decision-making stages may shape practice decisions and trajectories of these learners, including scope of practice and academic involvement [12, 14]. Advice can also prepare trainees for the realities of managing a practice, readying them for making logistical, fiscal, and human resource decisions [15]. In this study, we examine the types of advice early-career family physicians and family practice residents would offer to new family medicine residents. Findings from this study may provide insight into the existing challenges in family medicine and how these challenges may be shaping practice intentions.

## Methods

### Study design and population

We analyzed a subset of qualitative interview data from a larger mixed-methods study (*Practice patterns among early-career primary care physicians [ECPC]*) which explored factors contributing to practice intentions of early-career family physicians and family medicine residents across three Canadian provinces: British Columbia (BC), Ontario (ON), and Nova Scotia (NS). The complete study protocol has been published previously [16].

Participants were recruited via provincial medical association newsletters, family medicine residency programme email lists, and social media (Twitter and Facebook). To be included in the study, participants had to be a current family medicine resident or a family physician who had completed their family medicine residency between 2008 and 2018 and were currently practicing. Only those practicing in BC, ON, or NS were eligible. Prospective study participants completed a demographic screening questionnaire to ensure they met the inclusion criteria for the study. This questionnaire (Appendix 1) also served to facilitate diverse purposeful sampling based upon previously identified characteristics (i.e., gender, rurality, specialization, practice/training location, practice type/model, relationship status, and whether they have dependents) [16]. During recruitment, 359 residents and family physicians completed the demographic screening questionnaire. To ensure maximum variation, of those who completed the questionnaire, 32 family medicine residents and 69 early-career family physicians were purposively selected to participate in the study based on their responses to the screening questionnaire. Interviews were completed with 31 of 32 family medicine residents and 63 of 69 early-career family physicians invited to participate across the three Canadian provinces. Seven interviews were declined due to scheduling conflicts, lack of response, or an undisclosed reason. Participants were offered an honorarium.

### Data collection

Semi-structured, ~ 1-hour, in-depth telephone interviews (Appendix 2) were conducted to understand the practice patterns and motivations of early-career family physicians and family medicine residents in BC, ON, and NS. Interviews were conducted by experienced qualitative interviewers (one per province), at a time suitable for participants, thereby engaging more geographically diverse interviewees with lower overall costs. Interviews were audio-recorded and transcribed verbatim, removing personally identifiable information.

### Data analysis

Data were coded according to a robust coding framework developed by three experienced qualitative research analysts and supported by the principal investigators for the study. For the purpose of this paper, thematic analysis was performed by two analysts on the code “advice given to residents,” which corresponded to the interview question; *“If you were mentoring a new family medicine resident*,* what advice would you give them about planning their career as a family physician?”* Relevant excerpts were reviewed and analyzed independently by two analysts (SW, LRM), who identified initial themes. Refinements were made to the initial themes through discussion among analysts (SW, RB, LRM) and three general themes, with multiple subthemes, were agreed upon. Themes were finalized through discussion and support of all authors (SW, RB, LRM, MRL, EGM). This study was approved by the Simon Fraser University (#H18-03291), University of Ottawa (#S-05-18-776), and Nova Scotia Health Authority research ethics boards (#1023561).

## Results

Out of the 94 participants from the ECPC study, 60 early-career family physicians and 30 family medicine residents (90 total; 30 from BC, 30 from NS, 30 from ON) shared advice they could offer to family medicine residents and were included in this analysis. Table 1 shows the demographic and practice characteristics of participants. Participants practiced in a variety of settings and models. Men and women were interviewed, most of whom were partnered, and some cared for dependents (primarily children). Participants of this study did not agree for their individual data to be shared publicly, so supporting data are not available.

**Table 1:** Participating family practice resident (n = 30) and early-career family physician (n = 60) demographics

		Family practice residents (n = 30)	Early-career family physicians (n = 60)	
Province	Nova Scotia	11 (36.7%)	19 (31.7%)	
	Ontario	10 (33.3%)	20 (33.3%)	
	British Columbia	9 (30.0%)	21 (35.0%)	
Dependents	Yes, child(ren)	6 (20.0%)	31 (51.7%)	
	Yes, adult(s)	1 (3.3%)	2 (3.3%)	
	Yes, both	2 (6.7%)	1 (1.7%)	
	No	21 (70.0%)	26 (43.3%)	
Gender	Man	13 (43.3%)	26 (43.3%)	
	Woman	16 (53.3%)	33 (55.0%)	
	Unreported	1 (3.3%)	1 (1.7%)	
	Other	0 (0.0%)	0 (0.0%)	
Practice Setting*	Inner city	9 (30.0%)	15 (25.0%)	
	Urban/suburban	17 (56.7%)	32 (53.3%)	
	Small town	9 (30.0%)	16 (26.7%)	
	Rural	12 (40.0%)	24 (40.0%)	
	Remote	3 (10.0%)	8 (13.3%)	
Current Year of Residency	PGY1	13 (43.3%)	***N/A***	
	PGY2	16 (53.3%)	***N/A***	
	No data	1 (3.3%)	***N/A***	
Last Year of Residency	2010–2012	***N/A***	12 (20.0%)	
	2013–2015	*** N/A***	18 (30.0%)	
	2016–2018	*** N/A***	30 (50.0%)	
Medical School Graduation Location	Canada	21 (71.0%)	45 (75.0%)	
	Outside of Canada	9 (30.0%)	14 (23.3%)	
	Unreported	0 (0.0%)	1 (1.7%)	
Payment Type*	Blended	***N/A***	5 (8.3%)	
	Capitation	***N/A***	5 (8.3%)	
	Fee-for-service	***N/A***	44 (73.3%)	
	Salary	***N/A***	16 (26.7%)	
	Service Contract	***N/A***	14 (23.3%)	
	Sessional or per diem or hourly	***N/A***	27 (45.0%)	
	Other	***N/A***	1 (1.7%)	
Practice Model*	Group	22 (73.3%)	38 (63.3%)	
	Interprofessional Team	17 (56.7%)	26 (43.3%)	
	Solo	7 (23.3%)	9 (15.0%)	
	Other	1 (3.3%)	16 (26.7%)	
Practice Type After Residency	Comprehensive	12 (40.0%)	42 (70.0%)	
	Focused	9 (30.0%)	10 (16.7%)	
	Special Interest	8 (26.7%)	9 (15.0%)	
	Other	1 (3.3%)	4 (6.7%)	
Relationship Status	Single/divorced/separated/widowed	14 (46.7%)	22 (36.7%)	
	Married/common-law/life partner	16 (53.3%)	36 (60.0%)	
	Unreported	0 (0.0%)	2 (3.3%)	

*Some totals are higher than the sample size due to participants working in multiple locations/falling into multiple categories

Family physicians and residents provided many pieces of advice to potential family medicine residents. We identified three themes in the data: (1) advice on the importance of having diverse practice experiences; (2) advice on the unanticipated aspects of family medicine; and (3) advice on taking care of yourself while practicing family medicine.

### Theme 1. Advice on the importance of having diverse practice experiences

Participants who were early-career family physicians and residents emphasized the importance of experiencing a diversity of practice settings and fields. A family physician advised that residents should spend time ***“test[ing] the waters****”* and finding *“where you feel that you fit and you’re happy.”* (Family Physician, BC). As one participant explained:

*“I’d tell them to do as many electives in different places and experience as many different types of clinics and types of practices that they can… I never would have thought that I could be doing what I was doing. I sort of thought buying into a practice was sort of your only option… And I honestly tell people… don’t commit to something right away… there are so many opportunities out there that you’re not exposed to.”* (Family Physician, BC).

Several participants recommended that residents should ***locum first*** to gain experience across a variety of family medicine models, communities, and specialties. By locuming, new physicians can *“get the sense of how you want to schedule your day… You’re working with different types of administrators. You’re not having to work with your own administrators. So*,* you can figure out who you want to hire in a secretary or if you need more than one secretary… you get to see what it’s like either in a collaborative or a solo practice… see different areas*,* get different experiences as a working physician.”* (Family Physician, NS).

Locum work can provide new family physicians with the opportunity to *“try”* out potential future practice locations *“before putting roots down”* (Family Physician, NS). A family physician said that residents should know *“… it’s okay to locum and try out different types of models because at the end of the day*,* I hope that everyone can find the passion to do what they do in a model where they love how they get to practice medicine.”* (Family Physician, ON).

Interviewees advised that residents seek variety in areas of practice such as emergency and obstetrics and experience practicing in rural areas, where one can *“really see what the breadth of family medicine is”* (Resident, ON). Experience in other areas of medicine can offer family physicians *“flexibility”* in their practice. As a resident said, *“…you can work in a diverse number of environments… plus the operating room*,* palliative*,* long-term care*,* geriatrics… it’s so diverse… you just have to find as many opportunities as possible and build sort the practice you want…”* (Resident, ON).

### Theme 2. Advice on the unanticipated aspects of family medicine

#### The business of family medicine

Participants would advise residents on areas of knowledge not necessarily taught in the formal medical school curriculum but represented the realities of working as a family physician. Family physicians and residents noted a dearth of formal education about ***the “business” of family*** medicine and described situations where they learned by doing. Advice from early-career family physician participants in our study emphasized that new family physicians need to expose themselves to *“lots of learning around billing and management”* (Family Physician, NS) to better perform administrative tasks revolving around their future practices (e.g., billing, insurance, contract negotiations, starting financial planning early, get occupational health and safety training, and learn how to hire and fire staff). Billing is a necessary part of family medicine for fee-for-service physicians, but is not formally taught in medical school or postgraduate training. As one participant stated, *“No one taught me how to bill. That was a disaster – learning how to do that.”* (Family Physician, NS). Advice was given to *“… talk to preceptors that you work with and see … how do they handle hiring and firing people … how do they schedule … the logistics of being a family doctor”* and *“know who are support people are and who can advocate for you.”* (Resident, NS).

#### Realities of working under different models

Interviewees not only detailed that learning the administrative duties of a family physician is critical for effectively running their family medicine practice, but they also emphasized the difficulties and limitations that come with working within the current payment and practice models offered. For instance, interviewees discussed how the business side of family medicine intersected with their well-being. For example, new family physicians might need to create time for vacation when working in solo practice, but “*You need to find somebody to cover for you in that kind of model. So yeah*,* I don’t know that I can fully endorse that kind of work. But I think it is the most personally and professionally valuable”* (Family Physician, BC). Another interviewee suggested that new family physicians should consider how pay might influence work-life balance: *“I make twice as much per hour [working in emergency medicine] as working in a clinic. So*,* I can work half as much and have time for myself and my wife or kids or whatever’s in the future.”* (Family Physician, BC).

Not all interviewees were responsible for their own clinic, but many provided advice about the business of family medicine. Early-career family physician and resident participants in our study advised that physicians must be careful about the contracts they sign and the agreements they make. As one participant explained:

*“… avoid committing yourself to any contracts … I see so many new grads being taken advantage of all the time … they’re basically taking advantage of new grads who don’t have that knowledge.”* (Family Physician, BC).

Because it can be challenging to navigate the business of family medicine, a resident advised that other residents should *“… not just jump into the first offer that you’re given … you want to make sure that you’re not being over-worked. You want to make sure that you’re being compensated properly… aware of your call schedule… to know who your support people are, or who can, advocate for you. So*,* whether that’s [your provincial professional association]*,* whether it’s other physicians.”* (Resident, NS).

#### Relevancy of and support for family medicine

Family physicians and residents recommended that new residents have an *awareness of the relevancy of, and support for, family medicine*. Some interviewees voiced their frustration with the lower level of support family physicians receive, describing their profession as *“eroding”* and that residents should consider whether they want to do family medicine. As one participant explained, “*I could see it becoming less and less relevant. You know*,* being a generalist… That’s what I’m afraid of… So*,* I think I would tell them to have a back-up plan… a different skillset in medicine.”* (Family Physician, NS). Thus, to prevent the erosion of the family medicine profession and ensure it remains relevant, participants were urged to *“keep advocating for fee parity and improvements in family medicine”* (Family Physician, BC).

#### Complexity of family medicine

Furthermore, participants often discussed the ***high and increasing complexity of family medicine.*** Interviewees described how family physicians are responsible for caring for increasingly complex patients “*due to the [family physician] shortage… or the long timelines to get people into specialists.”* (Family Physician, NS). Because of the perceived growing expectations of family physicians, an interviewee advised that residents should *“go easy on yourself”* as there is a *“tendency of that [frustration] in family medicine because all the problems always come back to us*,*”* suggesting that once specialists have exhausted all of their options, the onus falls back on the family physician to decide *“now what are you going to do about it?”* (Family Physician, NS).

Participants also discussed important considerations about working with patients. As one interviewee described, *“… don’t… under-estimate… a patient’s knowledge of themselves even if it doesn’t fall into a guideline”* (Family Physician, NS), going on to imply the importance of considering the patients’ preferences in treatment.

As one participant described, the medical complexity of family medicine requires a flexible schedule, with consideration of patients with urgent or emergent needs, *“… there’s lots of surprises that come in in family medicine… you have to allow a little bit of flexibility in the schedule for urgent people you need to fit in*,* or people that come in with chest pain*,* or suicidality*,* or things like that. So*,* it’s teaching around… being flexible and giving people the time when they need it. But also teaching residents and learners that sometimes you just have to set down some ground rules with patients for their own benefit sometimes.”* (Family Physician, NS).

#### Importance of lifelong learning

Participants also emphasized the importance of ***lifelong learning*** in family medicine, sharing that *“… the minute that I think that I know everything about a subject is probably when I do something that I don’t mean to do and potentially harm a patient.”* (Family Physician, ON).

One participant advised future residents to “*expect change throughout your career… [w]hether that’s government changes*,* whether it’s the advent of AI and technology…”* (Family Physician, ON).

#### Perceived need to specialize within family medicine

Finally, not only were family medicine residents advised to be flexible and expect change throughout their careers, but participants also urged residents to ***specialize within an area of family medicine to stay afloat***. As a resident explained, *“I think the nature of family medicine is changing*,* and increasingly so*,* there are less and less true general practitioners. And so*,* if you don’t carve something out that you’re interested in*,* I think you kind of get lost in the shuffle”* (Resident, BC). Participants recommended that residents narrow their practice into a subspecialty like *“sports medicine or addictions”* (Family Physician, BC) and that residents *“…could apply for enhanced training skills or a plus one program to help develop those skills and make them more competitive after they’re done their training”* (Resident, NS).

### Theme 3. Advice on taking care of yourself while practicing family medicine

Participants offered several pieces of advice for residents to understand **how to take care of themselves in family medicine**. Of particular concern to participants was ***preventing burnout.*** Strategies for avoiding burnout included not *“jump[ing] right into a practice”* (Family Physician, BC), *“find[ing] a niche… Something you can do to get a good balance in your career and so you don’t burn out.”* (Resident, ON), and *“… guarding… personal and private time.”* (Family Physician, NS).

Many participants suggested that new family medicine residents need to ***prioritize work/life balance***. Family physician and resident participants discussed how general, full-time family medicine can lead to little personal flexibility and dissatisfaction with work and burnout. As a participant advised, *“I would tell them to design their life first and then find an area within family medicine*,* whether that’s clinic or otherwise*,* that will let them actually live the life they want. Because if you just sign up to be a doctor first*,* you can work endlessly and not have any time for yourself”* (Family Physician, BC).

Many participants discussed difficulties managing work and personal considerations and how poor balance in these areas can result in physicians leaving the profession. Participants provided advice for residents about planning for the future, with several participants suggesting that new residents should build their practice starting smaller, including core interests and then *“build from that. If you find you have room in your life*,* then add the second or third thing. Because it’s a lot easier to build up than it is to say no.”* (Family Physician, NS). Similarly, some participants advised that residents envision their personal goals and *“work backwards.”* As this participant suggested, *“picture where you want to be in 10 to 20 years*,* and then kind of plant the seeds.”* (Family Physician NS). Having a mentor or role model may help with this: *“… find people whose work-life balance reflects your own values. And then strive to follow a similar path.”* (Family Physician, BC).

Overall, participants advised that residents should ***find personal satisfaction in their work*** and *“end up with a career that you are truly passionate about and that you love”* (Resident NS). As previously mentioned, there was a substantial amount of advice provided on obtaining experience in a variety of areas. One participant advised that residents should *“… understand… the aspects of the work that you enjoy the most. And then you can use that to guide where you end up working. So*,* do you like having longitudinal relationships? Do you like quick diagnostics? Do you… like to do procedures? Do you like the complexity of working in resource-limited spaces?”* (Family Physician, BC). As participants described, it is essential that residents spend time finding what they like and dislike about their job because *“how am I going to be able to provide the care that I want to be able to provide to my patients without feeling angry or bitter or whatever it is about the system?”* (Family Physician, ON) and *“no one’s going to give you a medal at the end of 30 years for making yourself miserable.”* (Family Physician, BC).

### Subtheme 1. Advice comparison between providers, provinces, and genders

Responses from both residents and early-career family physicians were examined showing many similarities in the advice that they would give incoming residents. Common themes include finding work-life balance, being open-minded, becoming a mentor, finding self-fulfillment, being aware of pre-mature commitment, the need for practice specialization, importance of patient advocacy, and preventing burnout. Early-career family physicians were more likely to emphasize the importance of understanding practice logistics and achieving job security.

Between the three provinces, the advice participants would give are similar, with NS and ON emphasizing the importance of practice logistics and job security more than BC. Otherwise, all provinces gave similar advice on finding work-life balance, being open-minded, becoming a mentor, finding self-fulfillment, being aware of pre-mature commitment, the need for practice specialization, importance of patient advocacy, and preventing burnout.

Responses from both genders of participants showed commonality in discussing advice on finding work-life balance, being open-minded, becoming a mentor, finding self-fulfillment, being aware of pre-mature commitment, the need for practice specialization, the importance of understanding practice logistic, and preventing burnout. Male providers emphasized more about the importance of patient advocacy and achieving job security.

## Discussion

The advice early-career family physicians and family medicine residents would offer to new family medicine residents provides insight into not only the present challenges and opportunities in family medicine, but also how these factors influence their decision-making and adaptation processes in clinical practice. We found that the advice of early-career family physicians aligned with previous research, as we identified themes within our interviews such as challenges of staff management, coping with burnout, and the increased responsibility of family physicians to care for an increasingly complex patient population [9, 12, 14]. Importantly, our research provides new insight into the reasoning behind the these themes, including the perceived necessity for specialization within family practice, need for readiness in advancing technologies and health informatics, and advocacy for structural changes such as remuneration parity. Last, we examined differences between residents and early-career physicians. While themes of advice were similar, more emphasis was placed on practice logistics and job security by early-career physicians than residents.

Our first overarching theme of need for seeking diverse experiences in family medicine has previously been identified with fewer family physicians committing to and offering comprehensive primary care [13], and a greater number of family physicians are working under a specialized scope of practice, partitioning their practice into specific fields of interest, such as obstetrics, surgical assist, small procedures, and emergency medicine [11, 13, 16, 26, 27]. Our findings add to the current literature by demonstrating the reasoning behind this trend of specialized rather than generalized family practice; such that, family physicians may be doing this to remain competitive with respect to pay, reduce burnout with regards to schedule flexibility and evade contractual agreements that try to take advantage of new graduates [8, 13]. The insight gained from this advice allows health system planners and decision-makers to understand the factors considered by family physicians in how they practice and address the concerns in their policy-making, thereby attracting more family physicians to practice comprehensive clinic care.

We secondly demonstrated that family physicians and residents face a practice environment that poses challenges not only with clinically complex patients [23–25] but also developing a detailed understanding of practice logistics and business [20]. Our participants not only identify the gaps in education on financial planning, technological advances, and compensation, but strategies in which they take to mitigate these challenges and optimize clinical care for their patients. For instance, providers can take advantage of variety of scheduling, areas of focus, and practice models [17]. A novel finding in our study shows an emphasis on seeking preceptors that act as mentors in learning business management. This demonstrates an important wealth of knowledge on the administrative practice of family medicine, as formal curriculum in such topics are not concretely implemented in training [15, 16, 21, 22]. Finding mentorship from preceptors is especially important as our results demonstrate that residents are less likely to be concerned about practice logistics and job security than staff physicians. In brief, participants demonstrate that challenges endured by family physicians can be explored through taking advantage of practice flexibility and informal mentorship. Formal education may need to be more agile to train new cohorts of family physicians in a changing primary care landscape.

Family physicians and residents’ well-being and burnout prevention is a commonly identified theme in previous literature [14, 18, 19]. Interestingly, our interviewees recommended that in order to prevent burnout, new family physicians ought to focus on both structural and personal strategies such as start their career with a part-time practice and build up from there, designing their practice around the type of lifestyle they wish to have and ultimately practicing the aspects of family medicine they genuinely enjoy and value. However, participants noted that the current capacity of organizational and funding models available for family physicians limits their opportunities. A specific example includes being compensated fairly for their practice, with participants detailing the difficulties of specific family medicine remuneration models, such as the inflexibility of taking vacation time in a solo fee-for-service practice, or how specialized compared to general family practice is compensated relatively higher. This is supported by previous research, which suggests that fee-for-service remuneration models may discourage the practice of comprehensive family medicine [13]. Parity in pay may help family physicians feel more valued within the health system and encourage them to provide primary care services that are desperately needed within the Canadian health system [20]. It is evident that strategies to combat physician burnout is limited without policy changes to adequately fund comprehensive family practice.

In all, our study confirms previous research which found that the need for specialization, clinical complexity, burnout, and job logistics amalgamate, forming ongoing concerns for family medicine residents [14]. Not only does it emphasize these themes, it identifies motives, strategies, and reasoning behind the practice patterns and trends of current family physicians and how they adapt to current challenges and opportunities. While participants shared many positive aspects of family medicine, they focused significantly on the perils and challenges of practicing family medicine. When we are in a time of needing to increase the number of family physicians to meet the primary care needs of patients across the country, it may be important to reflect on how more experienced family physicians influence trainees away from family practice and the need to advocate for improvements in primary care such as revising formal curriculum to include non-clinical aspects of practice, parity in remuneration, and mentorship supports for new family physicians. Individual-level interventions are not enough to counter system-level challenges. Systemic interventions will be needed if system decision-makers wish to encourage greater involvement in comprehensive family medicine [26, 28–30].

## Conclusion

Advice from early-career family physicians and senior family medicine residents to new family medicine residents emphasized the importance of taking advantage of early-career opportunities, understanding the realities and complexities of modern family medicine, and advocating for personal well-being and satisfaction. Our results also revealed the strategies and decision-making behind the current family medicine curriculum and practice trends. Systemic intervention is needed to support family physicians throughout their entire careers to allow them to practice in a way that is personally and professionally fulfilling while supporting their lifelong learning in an ever-changing field.

### Electronic supplementary material

Below is the link to the electronic supplementary material.

Supplementary Material 1

## Data Availability

The datasets supporting the conclusions of this article are included within the article.

## Abbreviations

BC: British Columbia
NS: Nova Scotia
ON: Ontario
CME: Continuing Medical Education
