# Supplementary material for: Advice to future family physicians: findings from qualitative interviews with family medicine residents and early-career family physicians
Source: BMC Med Educ. 2024 Aug 20;24:897. doi: 10.1186/s12909-024-05882-5 (PMC11334307; doi:10.1186/s12909-024-05882-5)
Supplement: Supplementary file 1 — Supplementary Material 1 [file 12909_2024_5882_MOESM1_ESM.docx]

# Appendices

## Appendix 1: Screening Surveys for family physicians and residents

### Resident Family Physician Survey

1. What is your gender?

Female

Male

Non-binary / third gender

Prefer to self-describe

Prefer not to answer

1. What is your current relationship status?
    Single/Divorced/Separated/Widowed
    Married/Common-law/Life Partner
    Other, please specify: _______________

Prefer not to answer

1. Do you have any dependents (e.g., child, parent, grandparent)?
    Yes – child(ren)

Yes – adult(s)

Yes – both

No

1. Is there another person (non-dependant adult) in your life who influences decisions you make about how and where you practice?

Yes  No

1. Where did you graduate from medical school?
    Canadian Medical Graduate
    International Medical Graduate
2. Which of the following best describe the community(ies) in which you are doing your residency?

Please select all that apply.
 Inner city
 Urban/suburban
 Small town
 Rural
 Remote
 Mixture of environments

1. Which of the following best describes the organizational model(s) in which you practice?
   Please select all that apply.
    Solo practice
    Group physician practice
    Interprofessional team-based practice
    Other, please specify: _________________
2. Which best describes the kind of practice you intend to have after your residency?
    Comprehensive practice
    Special interest practice (less than 50% of the time on specialized care)
    Focused practice (50% or more of the time on specialized care)
    Other, please specify: _________________

### Early-Career Family Physician Survey

1. What is your gender?

Female

Male

Non-binary / third gender

Prefer to self-describe

Prefer not to answer

1. What is your current relationship status?
    Single/Divorced/Separated/Widowed
    Married/Common-law/Life Partner
    Other, please specify: _______________

Prefer not to answer

1. Do you have any dependents (e.g., child, parent, grandparent)?
    Yes – child(ren)

Yes – adult(s)

Yes – both

No

1. Is there another person (non-dependant adult) in your life who influences decisions you make about how and where you practice?

Yes  No

1. Where did you graduate from medical school?
    Canada
    Outside of Canada
2. Which of the following best describe the community(ies) in which you predominantly practice?
   Please select all that apply.
    Inner city
    Urban/suburban
    Small town
    Rural
    Remote
    Mixture of environments
3. Which of the following best describes the organizational model(s) in which you practice?
   Please select all that apply.
    Solo practice
    Group physician practice
    Interprofessional team-based practice
    Other, please specify: _________________
4. Which of the following best describes your practice?
    Comprehensive practice
    Special interest practice (less than 50% of the time on specialized care)
    Focused practice (50% or more of the time on specialized care)
    Other, please specify: _________________
5. Which of the following are methods by which you receive payment?
   Please select all that apply.
    Fee-for-service
    Salary
    Capitation
    Sessional/per diem/hourly
    Service contract
    Blended
    Other, please specify: _________________
